# msDNA: A “Wake‐on Sensor” for Defense Effectors in Prokaryotic Retron Systems

**DOI:** 10.1002/mco2.70758

**Published:** 2026-05-10

**Authors:** Hongbiao Ran, Min Wu, Ping Lin

**Affiliations:** ^1^ Biological Science Research Center, Chongqing Technology Innovation Center of Breeding, Academy for Advanced Interdisciplinary Studies, Southwest University Chongqing China; ^2^ Wenzhou Institute, University of Chinese Academy of Sciences, Zhejiang Wenzhou China; ^3^ Department of Medicine Harvard Medical School, Brigham and Women's Hospital Boston Massachusetts USA

1

In two back‐to‐back recent papers published in *Molecular Cell* [1, 2] revealed how the retron Eco8 system restricts phage infection. Its “sensor”, msDNA, rapidly activates effector activity upon sensing phage‐specific SSB proteins. Although retron systems were systematically reported in 2020 to restrict bacteriophages [3], the interplay between msDNA and other components remains largely unclear. These new findings redefine msDNA's role in retron immunity and offer insights for developing msDNA‐based gene‐editing tools.

The “arms race” between bacteria and bacteriophages has persisted for billions of years. Like the human immune system, bacteria have evolved sophisticated defense mechanisms. Among them, the retron system typically comprises a reverse transcriptase (RT), a retron‐specific non‐coding RNA (ncRNA), and a variable effector protein component. The ncRNA contains inverted repeats at the 5' end of msrRNA and the 3' end of msdRNA, marked by a conserved guanosine residue (branching G) that primes reverse transcription. RT uses msdRNA as a template to synthesize cDNA. Reverse transcription halts before the msrRNA segment, and the template RNA is degraded by RNase H but the msrRNA. A covalent 2'‐5' phosphodiester bond forms between msdDNA and msrRNA at the branching G [3]. This msDNA (multisubstrate single‐stranded DNA) hybrid typically adopts a stem‐loop structure, and its distinctive conformation and specific targeting have sparked great interest among biologists and biomedical scientists, driving efforts to repurpose retron systems for genome engineering.

Given that RT lacks domains beyond those required for RNA‐dependent polymerase activity, an effector with cytotoxic or clearance capability is essential for the retron to function as an antiphage system. In the retron Eco8 system [1, 2], RT, msDNA, and the OLD (overcoming lysogenization defect) effector assemble into a symmetric and stable 4:4:4 super‐complex. Four OLD protomers form a tetramer arranged as a dimer of dimers in the core, and four msDNA‐RT subcomplexes, formed by electrostatic interactions between msDNA and RT, are recruited to and extensively engage with the OLD nuclease at the interface of its ATPase and Toprim domains (Figure 1). Upon bacteriophage infection, the Eco8 complex transitions to an active conformation following binding of phage single‐stranded DNA‐binding (SSB) protein. SSB does not interact with OLD, but instead binds msDNA, releasing the nuclease active site of OLD effectors through stem‐loop shortening or unwinding. Meanwhile, SSB displaces msDNA on the Eco8 super‐complex by pulling out its embedded 3' end, thereby disrupting the interaction between OLD nuclease and the msDNA‐RT subcomplex. SSB is therefore proposed to act as the direct trigger that activates the OLD nuclease effector: its binding destabilizes the apo‐state super‐complex, promotes reorganization, and creates binding sites for effectors and heterologous DNA. These findings support a hypothetical model in which msDNA functions as a fishnet‐like molecular latch during Eco8 activation: upon phage invasion, the taut msDNA unravels and releases the encapsulated effectors, thereby activating the system to counteract the intruder (Figure 1).

**FIGURE 1:**
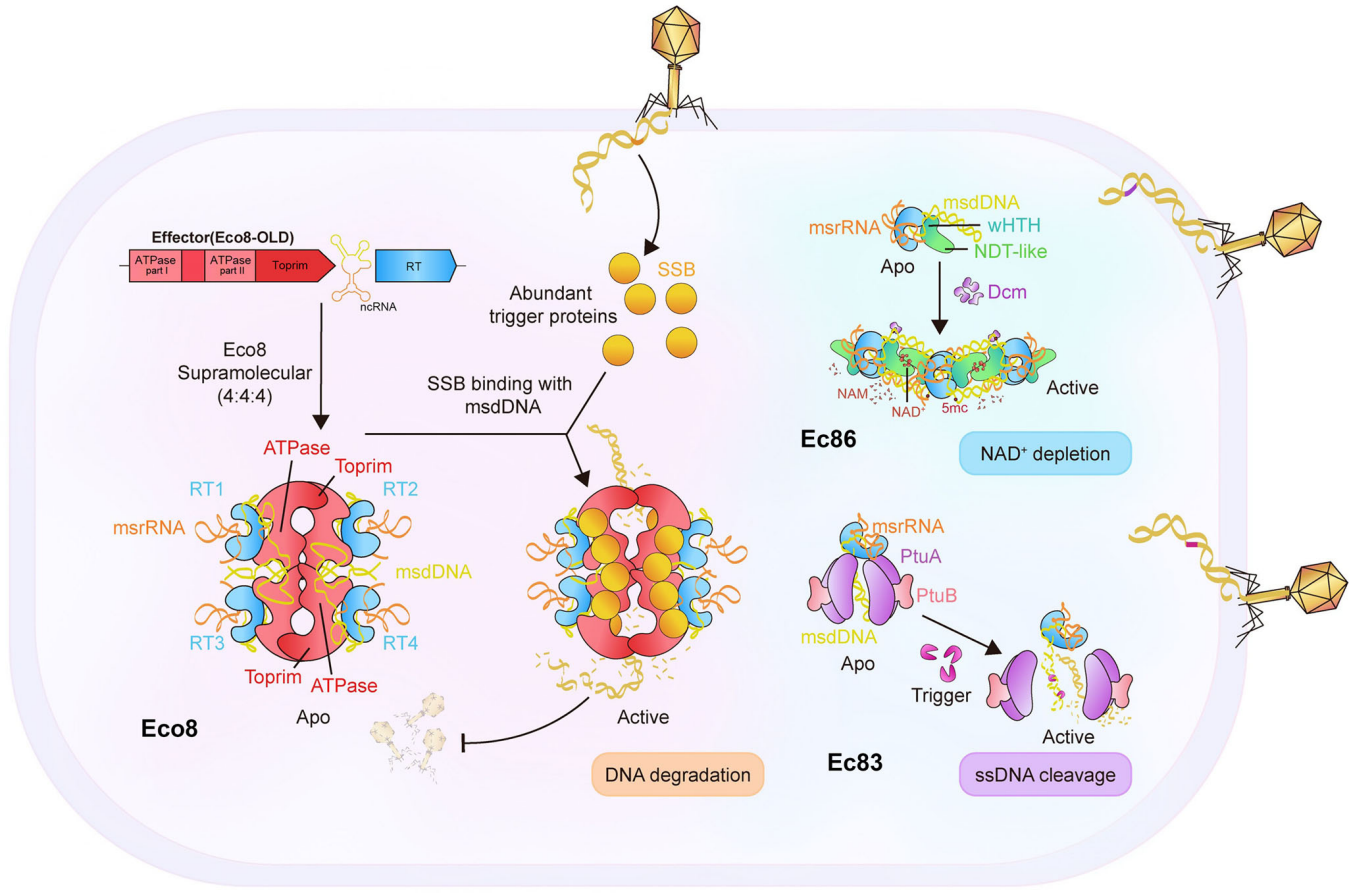
The regulatory role of msDNA in retron systems. Under normal physiological conditions (left), Eco8 components assemble into a 4:4:4 super‐complex. The msrRNA and segments of msdDNA encircle the Eco8 RT (reverse transcriptase) through electrostatic interactions, serving as structural linkers that enhance the stability of the complex formed by RT and OLD (overcoming lysogenization defect) protomers. Upon phage infection, phage SSB (single‐stranded DNA‐binding) protein is markedly upregulated and associates with msDNA within the super‐complex, pulling a substantial portion of msdDNA out of the channel between RT and OLD nuclease. This disruption of msDNA's stabilizing role leads to disassembly or loosening of the super‐complex, exposing the active sites of the OLD nuclease effectors. The steady state of msDNA similarly governs the activation‐dormancy switch of effectors in other retron systems (right), suggesting that msDNA plays a general regulatory role in retron systems.

Yuan et al. [1] and Yu et al. [2] shed light on the msDNA as an SSB target that influences retron super‐complex stability in its apo state, but local resolution at the SSB‐msDNA interface limits detailed definition of the contacts, leaving it unclear whether physicochemical features of SSB components drive msDNA deformation. On one hand, whether this interaction is direct or indirect remains uncertain. The speculation that virus‐induced ionic changes contribute to destabilization, given the electrostatic association of msDNA within the retron super‐complex [2, 4, 5], warrants further investigation. On the other hand, SSB binding disrupts extensive pre‐existing interactions, suggesting it binds msDNA with higher affinity and stability. SSB likely binds the msDNA stem‐loop (DSL) via a tetramer, as an average of four SSB molecules associate with each msDNA‐RT subcomplex in Eco8, and the DSL is known to inhibit effector active sites across retron systems [2, 4, 5]. It also remains to be seen whether molecular mechanisms differ among various phage SSBs. Finally, a substantial number of trigger proteins (16 SSBs total) is required to stimulate the Eco8 system, implying activation occurs at a late stage of phage invasion. Thus, how the retron system cooperates with primary immune components merits further exploration.

In the Eco8 super‐complex, the msdDNA segment engages minimally with RT but robustly with OLD nuclease via its DSLa [1, 2], stabilizing complex formation (Figure 1) in a manner reminiscent of msdDNA architecture in Ec86 and Ec83. In Ec86, two RTs enveloped by msDNA form a scaffold, with its msdDNA segment (featuring a long‐arm stem‐loop), extending outward to engage effectors extensively. These interactions promote effector dimerization within the interspaces between two DSLs, a configuration critical for complex stability and phage defense [4]. In Ec83, msDNA similarly wraps around RT electrostatically, while its DSL, positioned away from RT, recruits two effector subcomplexes to form a stable Ec83 super‐complex [5]. Despite this conserved assembly role, msDNA employs distinct release mechanisms upon phage infection: in Ec86, the DSL is modified by a phage‐encoded Dcm‐like methyltransferase, triggering effector release and NAD^+^ degradation [4]; in Ec83, msDNA is degraded upon phage stimulation, leading to super‐complex disassembly or ssDNA cleavage [5]. These findings illustrate that msDNA acts as a direct regulator of effector activation in retron systems, suggesting that targeting msDNA to destabilize the complex or awaken “dormant” effectors represents a conserved activation strategy that may reflect specific recognition of bacteriophages.

Unlike most msDNAs [3, 4, 5], Yu et al. [2] reported that the 5' repeat sequence of msrRNA in Eco8 is degraded via an unknown mechanism, and the mature msDNA contains extra single‐stranded regions beyond its stem‐loops. Notably, this was not observed by Yuan et al. [1]. Although mature msDNA sequences tolerate variation without compromising phage defense [4, 5], the repeat arm and loop structures remain critical for msDNA production in retron‐based gene editing tools, prompting us to hypothesize that that the unique sequence in Eco8‐msDNA may be linked to its biosynthesis. Moreover, each Eco8 subcomplex requires an average of four SSB proteins to trigger supramolecular rearrangement, underscoring the importance of component stoichiometry for system activation. Finally, retron‐Eco8 exhibits distinct phage resistance profiles [1, 3], likely arising from structural differences in msDNA, given the high specificity of RT and effector functions. These directions will be interesting topics for future investigation.

The retron system stands out as one of the most promising prokaryotic immune systems for genome editing applications. The present studies further demonstrated that msDNA acts as a molecular switch governing effector activity, offering a new perspective on the regulatory mechanism of retron systems. Nevertheless, the relationship between msDNA sequence or stem‐loop structure and the specificity of exogenous element recognition is still unclear. Elucidating how flexible regions contribute to target recognition and binding is critical for improving the specificity of ncRNA or msDNA modifications in retron‐based tools. Meanwhile, many retron systems in nature remain to be characterized, which is especially true for effectors with predicted functions distinct from NAD hydrolases and nucleases, such as transmembrane proteins, cold‐shock DNA‐binding proteins, and phosphoribosyltransferases [3]. Harnessing these systems for genome editing requires a deeper understanding of the molecular mechanisms underlying pathogen resistance, posing challenges for both the discovery of novel retrons and the elucidation of their functions. Conceivably, advancements in system identification methods and exploration of diverse bacterial species may reshape current paradigms of msDNA regulation, while also offering a highly competitive and promising direction for future research in autonomous gene editing, particularly when coupled with effectors exhibiting specific cleavage activity.

## Author Contributions

Hongbiao Ran wrote the manuscript and prepared the figure. Min Wu and Ping Lin discussed and revised the manuscript. All authors have read and approved the final manuscript.

## Finding

This research was funded by National Natural Science Foundation of China (32470022 and 82470005) and Chongqing Graduate Student Research Innovation Project (CYB240149).

## Ethics Statement

The authors have nothing to report.

## Conflicts of Interest

The authors declare no conflicts of interest.

## Data Availability

The authors have nothing to report.
